# The Health Effects of Dietary Supplements

**DOI:** 10.1155/2022/9851048

**Published:** 2022-05-31

**Authors:** Alessandra Durazzo, Daniel Dias Rufino Arcanjo, Massimo Lucarini

**Affiliations:** ^1^CREA-Research Centre for Food and Nutrition, Via Ardeatina 546, Rome 00178, Italy; ^2^Laboratory of Functional and Molecular Studies in Physiopharmacology (LAFMOL), Department of Biophysics and Physiology, Federal University of Piauí, 64049-550 Teresina, Piauí, Brazil

Traditional medicinal plants and herbs are commonly used for health purposes and have been extensively studied [1–4]. Nowadays, dietary supplements have received particular interest worldwide as they are valuable health tools in disease management [5–8]. They are relatively easy to use. Moreover, they are cost-effective when compared with chemical entities obtained from synthesis. Botanicals, one of the most emerging classes of dietary supplements, are made of herbs. They are also created by mixing different herbs from raw materials, from whole plants, or from certain parts. This includes flowering herbs, leaves, leaf exudates, fruits, berries, roots, and rhizomes.

Current methodologies enable us to isolate, standardize, and characterize fractions of medicinal plants with specific bioactivities. However, there is a need to investigate further methodologies. Nowadays, research focuses on new formulations and the health properties of dietary supplements. Moreover, dietary supplement research is progressively integrating multidisciplinary research approaches. Current and novel research includes emerging technologies such as nuclear magnetic resonance (NMR) spectroscopy, isotopic ratio mass spectrometry, multielemental analysis, fluorescence, near-infrared (NIR) spectroscopy, mid-infrared (MIR) spectroscopy, and mass spectrometry combined with chemometrics [9–12].

This Special Issue aimed at bringing together original research and review articles discussing our current knowledge of the health effects of dietary supplements. Multidisciplinary approaches, with particular focus on the investigation of the quality assessment and control of dietary supplements, have been explored into following topics: (i) isolation and quantification of natural products used for dietary supplements (e.g., standardized fractions and emerging technologies involving chemometrics); (ii) *in vitro* and *in vivo* research investigating the potential health properties of natural products used in dietary supplements; (iii) classification and categorization of dietary supplements; (iv) quality assessment and control of dietary supplements, with particular focus on the metrological approach.

In this context, Wu et al. [13] elucidated the role of cinnamic acid in amelioration of nonalcoholic fatty liver disease by suppressing hepatic lipogenesis and promoting fatty acid oxidation. A systematic review and meta-analysis of 10 randomized controlled trials on the improving effect and safety of probiotic supplements on patients with osteoporosis and osteopenia was carried out by Zeng et al. [14]. Yarizadeh et al. [15] studied the effects of omega-3 supplementation on resting metabolic rate in a systematic review and meta-analysis of clinical trials. On the other hand, edible *Cyanobacterium Arthrospira platensis* collected from the south Atlantic coast of Morocco was studied and proposed as a promised source of dietary supplements [16].

New functionalities of foods are also considered. For instance, the work of Salamatullah et al. [17] explored the bioactive properties of coffee beans, with particular regards to the effect of roasting. In another work, Salamatullah et al. [18] studied the effects of different solvents extractions on total polyphenol content, HPLC analysis, antioxidant capacity, and antimicrobial properties of peppers (red, yellow, and green *Capsicum annum* L.). Another example is given by Elhadef et al. [19] who studied pistachio hull extract as a practical strategy to extend the shelf life of raw minced beef: compared to synthetic antioxidants, the pistachio hull extract could be a clean-label alternative that can protect and enhance the quality of meat products. Song et al. [20] studied the biological functions of diallyl disulfide, a garlic-derived natural organic sulfur compound.

de Morais Lima et al. [21] studied the effects of “Bacuri” seed butter (*Platonia insignis* Mart.) on metabolic parameters in hamsters with diet-induced hypercholesterolemia: Bacuri seed butter at doses of 25 and 50 mg/kg/day has positive repercussions on the lipid profile, more precisely on plasma HDL-c and LDL-c, and additionally promotes reduction in the risk of atherosclerosis in hamsters.

All articles, part of this Special Issue, reflect new trends and promote new ideas for future collaborative network and infrastructure in perspective of update, and standardize the study approach and quality control, adding new information and sharing data.

We hope that the readers will find this Special Issue interesting and inspiring.

## References

[1] Santini A., Tenore G. C., Novellino E. (2017). Nutraceuticals: a paradigm of proactive medicine. *European Journal of Pharmaceutical Sciences*.

[2] Santini A., Novellino E. (2017). To nutraceuticals and back: rethinking a concept. *Foods*.

[3] Durazzo A., D’Addezio L., Camilli E. (2018). From plant compounds to botanicals and back: a current snapshot. *Molecules*.

[4] Daliu P., Santini A., Novellino E. (2019). From pharmaceuticals to nutraceuticals: bridging disease prevention and management. *Expert Review of Clinical Pharmacology*.

[5] Van Breemen R. B. (2015). Development of safe and effective botanical dietary supplements. *Planta Medica*.

[6] Dwyer J. T., Coates P. M., Smith M. J. (2018). Dietary supplements: regulatory challenges and research resources. *Nutrients*.

[7] Dwyer J. T., Saldanha L., Bailen R. (2021). Commentary: an impossible dream? integrating dietary supplement label databases needs, challenges, next steps. *Journal of Food Composition and Analysis*.

[8] Saldanha L. G., Dwyer J. T., Bailen R. A. (2021). Modernization of the national institutes of health dietary supplement label database. *Journal of Food Composition and Analysis*.

[9] Betz J. M., Rimmer C. A., Saldanha L. G. (2018). Challenges in developing analytically validated laboratory-derived dietary supplement databases. *Journal of Nutrition*.

[10] Singh H., Bharadvaja N. (2021). Treasuring the computational approach in medicinal plant research. *Progress in Biophysics and Molecular Biology*.

[11] da Silva R. F., Carneiro C. N., de Sousa C. B. (2022). Sustainable extraction bioactive compounds procedures in medicinal plants based on the principles of green analytical chemistry: a review. *Microchemical Journal*.

[12] Durazzo A., Sorkin B. C., Lucarini M. (2022). Analytical challenges and metrological approaches to ensuring dietary supplement quality: international perspectives. *Frontiers in Pharmacology*.

[13] Wu Y., Wang M., Yang T. (2021). Cinnamic acid ameliorates nonalcoholic fatty liver disease by suppressing hepatic lipogenesis and promoting fatty acid oxidation. *Evidence-Based Complementary and Alternative Medicine*.

[14] Zeng L., Yu G., Yang K., Wensa H., Chen H. (2021). The improving effect and safety of probiotic supplements on patients with osteoporosis and osteopenia: a systematic review and meta-analysis of 10 randomized controlled trials. *Evidence-Based Complementary and Alternative Medicine*.

[15] Yarizadeh H., Hassani B., Nosratabadi S. (2021). The effects of omega-3 supplementation on resting metabolic rate: a systematic review and meta-analysis of clinical trials. *Evidence-Based Complementary and Alternative Medicine*.

[16] Ennaji H., Bourhia M., Taouam I. (2021). Physicochemical evaluation of edible Cyanobacterium Arthrospira platensis collected from the south atlantic coast of Morocco: a promising source of dietary supplements. *Evidence-Based Complementary and Alternative Medicine*.

[17] Salamatullah A. M., Hayat K., Husain F. M. (2021). Effect of microwave roasting and extraction solvents on the bioactive properties of coffee beans. *Evidence-Based Complementary and Alternative Medicine*.

[18] Salamatullah A. M., Hayat K., Husain F. M. (2022). Effects of different solvents extractions on total polyphenol content, HPLC analysis, antioxidant capacity, and antimicrobial properties of peppers (red, yellow, and green (*Capsicum annum* L.)). *Evidence-Based Complementary and Alternative Medicine*.

[19] Elhadef K., Ennouri K., Fourati M. (2021). Pistachio hull extract as a practical strategy to extend the shelf life of raw minced beef: chemometrics in quality evaluation. *Evidence-Based Complementary and Alternative Medicine*.

[20] Song X., Yue Z., Nie L., Zhao P., Zhu K., Wang Q. (2021). Biological functions of diallyl disulfide, a garlic-derived natural organic sulfur compound. *Evidence-Based Complementary and Alternative Medicine*.

[21] de Morais Lima G., da Silva Brito A. K., de Farias L. M. (2021). Effects of “bacuri” seed butter (platonia insignis Mart.) on metabolic parameters in hamsters with diet-induced hypercholesterolemia. *Evidence-Based Complementary and Alternative Medicine*.

